# How Do Children Socially Learn from Narrative Fiction: Getting the Lesson, Simulating Social Worlds, or Dialogic Inquiry?

**DOI:** 10.1007/s10648-022-09667-4

**Published:** 2022-03-04

**Authors:** Luciano Gasser, Yvonne Dammert, P. Karen Murphy

**Affiliations:** 1 Bern University of Teacher Education, Fabrikstrasse 8, 3012 Bern, Switzerland; 2 The Pennsylvania State University, University Park, PA 16802 USA

**Keywords:** Literary education, Social development, Empathy, Moral reasoning, Classroom dialogue

## Abstract

Educators read narrative fiction with children not only to promote their literacy skills, but also to support their sociomoral development. However, different approaches strongly diverge in their explanations and recommended instructional activities. Informed by theoretical understandings of reader-text transactions, this integrative review presents three different conceptions about how children learn socially from narrative fiction. The first approach explains sociomoral learning through narrative fiction by children’s extraction and internalization of the text’s moral message. The second approach refers to children’s training of mindreading and empathy as they become immersed in a fictional social world and imaginatively engage with the fictional characters’ perspectives. The third approach focuses on children’s social reasoning development through engagement in argumentative dialogues with peers about the complex sociomoral issues raised in narrative fiction. The article aims to theoretically position a wide range of literary programs to clarify their psychological foundations as well as critically discuss their strengths and limitations.

Since the 1980s, philosophers of art have increasingly emphasized and refined the Aristotelian idea that narrative fiction has educational value for readers’ sociomoral development (Carroll, 2000; Nussbaum, 1985). According to this, narrative fiction represents a developmental context for cultivating sociomoral competences, such as perspective-taking, empathy, and contextualized moral judgments. Cognitive psychologists have argued that narrative fiction creates a kind of simulation that enables readers to relate to fictional characters and explore their perspectives from a safe distance, but with vivid intimacy, resulting in improved mindreading and empathy (Kidd & Castano, 2013; Mar, 2018b). Similarly, educators see many social benefits to reading narrative fiction with children (Alatalo & Westlund, 2019). For example, parents perceive shared book readings as opportunities to transmit moral knowledge and deepen the relationships with their children (Audet et al., 2008).

Although it might seem that researchers and educators strongly agree on the role narrative fiction plays in sociomoral development, a closer look reveals significant differences between the various approaches. In a comprehensive research program, the Center for History of Emotions at Berlin’s Max Planck Institute for Human Development studied how the education of feelings through children’s literature has changed in different national contexts over the last two centuries (Frevert et al., 2014). The results revealed that since the nineteenth century, due to a broad democratic movement, the didactic tone in children’s literature decreased, while less hierarchical and more egalitarian characterization of parent–child relationships increased. Young fictional characters were increasingly depicted as autonomous agents who search for their identity free from adult and religious norms. The decreasing mode of top-down socialization from adults onto children has also allowed for more complex analyses of peer relationships, including its darker sides with peer aggression and victimization. However, the historical analyses also showed that this process was nonlinear and heterogeneous, and that all periods included conflict.

More contemporary perspectives on the role of narrative fiction in sociomoral development are also characterized by conflicting trends. While “critical literacy” aims at encouraging young readers to question social conventions, power relationships, and institutional inequalities (e.g., Luke, 2018), literacy programs in traditional character education focus on culturally conforming behaviors (Kilpatrick, 1993). In the 1990s, book lists such as *The* *Moral Compass* (Bennett, 1995) highlighted the educational value of “classic stories.” These stories were expected to transmit universal morals to children to mitigate the negative effects of declining values in today’s society.

Given the conflicting viewpoints emanating from the psychological and educational literatures and the importance of understanding the role of narrative fiction in sociomoral development, we conducted an integrative review of these literatures. Our primary goal is to compare different psychological explanations and instructional approaches on sociomoral learning through narrative fiction. According to Cronin and George (2020), the main purpose of an integrative review is to “(…) provide insight on a topic by synthesizing knowledge across communities of practice” (p. 2). Therefore, we aim to integrate research that approached sociomoral learning from narrative fiction from fundamentally different perspectives. We abstract themes from the research literature and integrate them within a broader theoretical framework of reader stances (Rosenblatt, 1978). We thereby identify the implicit assumptions of each perspective and critically evaluate their theoretical arguments and empirical foundation. A summary of the abstracted themes and their synthesis is displayed in Table 1.

This integrative review systematizes the literature around the following questions: Do children socially learn from narrative fiction by extracting moral messages from texts that they internalize when reading is over? Do they build sociomoral competences through their imaginative and emotional engagement with the fictional characters’ perspectives while they are reading? What is the evidence for the idea that children develop sociomoral competences by engaging in critical thinking and argumentative dialogues with peers about narrative fiction? Drawing on Rosenblatt’s (1978) transactional theory as well as other influential works on reader-text interaction (e.g., Chinn et al., 2001), we present three conceptions about sociomoral learning through narrative fiction. From the *efferent stance*, children’s attention primarily focuses on the abstract moral propositions of narrative fiction. Sociomoral development is conceptualized as the continuous accumulation of these propositions, also referred to as “acquaintance approach” (Carroll, 2000). In contrast, from the *expressive* *stance*, the reader develops mindreading and empathy skills by becoming immersed in the story and imaginatively following the characters’ perspectives. However, these expressive experiences only reflect the raw material and starting point for a more critical and reflective engagement with narrative fiction (Chinn et al., 2001; Rosenblatt, 1995). From a *critical-analytic* *stance*, the readers’ attention focuses on the critical reflection of initial responses to narrative fiction by engaging in argument-based dialogues with peers that require children to legitimize their own perspectives while considering peers’ alternative perspectives (Murphy et al., 2014).

Before we systematize the literature on sociomoral learning through narrative fiction according to the three stances (efferent, expressive, and critical-analytic), we clarify the term *sociomoral competence* by referring to the three most important developmental psychological concepts: Theory of Mind (ToM), empathy, and social reasoning. We then describe each stance in a separate section. For each of these sections, we distinguish between research on *psychological assumptions* and *educational approaches*. Under psychological assumptions, we discuss the learning processes that are used to explain the effects of narrative fiction on sociomoral development, in particular, direct instruction (efferent), social simulation (expressive), and discursive learning (critical-analytic). Under educational approaches, we refer to intervention programs that recommend specific instructional activities related to the specific stance. Based on this integrative review, we suggest future directions for research on sociomoral learning through narrative fiction. This includes a discussion on how the different perspectives might complement each other. We also point to minority positions that received relatively little attention in the psychological or educational research.

Previous literature reviews either systematized the psychological literature (Mar, 2018b; Oatley, 2016) or the educational literature (Schrijvers et al., 2019b) and mostly reviewed the literature from within a specific “community of practice.” By integrating the psychological and educational literature within each of the three stances, we clarify the implicit psychological assumptions underlying specific educational approaches. We thereby hope to provide guidance for researchers and practitioners to position a wide variety of different psychological and instructional approaches and assess their explanatory power regarding sociomoral learning through narrative fiction.

## Sociomoral Competence

We define sociomoral competence as the ability to take the perspective of and feel emotional concern for another person as well as engage in complex social reasoning that aims to balance conflicting interests. This definition builds on research on three developmental psychological concepts—ToM, empathy, and social reasoning. We justify the focus on these three concepts by their prominent role in the developmental literature as well as the literature on sociomoral learning through narrative fiction.

*Theory of Mind* (ToM) relates to children’s understanding of mental states, such as intentions, emotions, desires, and beliefs, which build the naïve psychology children use to explain and predict human behavior (for a review, see Wellman, 2010). The developing ToM indicates children’s increasingly sophisticated understanding of the subjectivity of mental states and the differences between the perspectives of self and others. In a scaling study including a battery of tasks covering different aspects of ToM, Wellman and Liu (2004) provided support for a developmental progression in 2- to 6-year-old children: Children first develop an understanding of the subjectivity of desires by judging that two persons can have different desires about the same object (e.g., one likes broccoli, the other does not). Children then increasingly understand that two persons can have different beliefs about the same situation. This development peaks in children’s insights about false beliefs, which are typically assessed by tasks on unexpected transfer. For example, children are told a story about a protagonist who places his sweets in location A; during his absence, the sweets are relocated from location A to B. Children are then asked to predict where the protagonist will look for the sweets. The task requires children to differentiate between their own true belief and the protagonist’s false belief. In addition, they need to understand that human behavior is not guided by real-world facts, but by our beliefs about them. Finally, children become aware that persons can display emotions other than those they really feel (e.g., smiling gratefully when receiving a disappointing present). During the elementary school years, children further refine their social understanding. For example, they develop an understanding of mixed emotions (e.g., feeling happy and sad at the same time) or ironic statements and complex forms of deception. In studies on the effects of narrative fiction on ToM in adults (e.g., Kidd & Castano, 2013), researchers often used the Reading the Mind in the Eyes Test (RMET; Baron-Cohen et al., 2001). In this test, participants see different pictures of the eye region and are asked to choose one of four words to best describe what the person in the photograph might be feeling or thinking.

The capacity to feel *empathy* strongly relates to ToM, but additionally includes an affective component. Empathy is defined “(…) as an affective response that stems from the apprehension or comprehension of another’s emotional state or condition and is similar to what the other person is feeling or would be expected to feel in the given situation” (Eisenberg et al., 2014, p. 84). Empathy can be distinguished from simple emotional contagion where the child is unaware of the source of his or her affective response (personal distress). Emotional contagion (e.g., reflexive crying when hearing another person crying) occurs in newborns as well as infants and it is associated with self-centered behaviors to relieve personal distress (Eisenberg et al., 2014). With the growth of language and social-cognitive skills in a child, empathic responses to others’ distress increase in complexity. In studies on the effects of narrative fiction on sociomoral learning, empathy is often assessed through self-reports on the Interpersonal Reactivity Index (IRI) developed by Davis (1983), which includes the subscales fantasy, perspective-taking, empathic concern, and personal distress. ToM and empathy both represent key sociomoral competences that relate to children’s or adolescents’ psychological and academic adaption (e.g., Dore et al., 2018; Eisenberg et al., 2014).

In contrast to empathy and ToM, *social reasoning* has been less often discussed in relation to literary education. We therefore elaborate developmental trends in social reasoning in some more detail. Social reasoning addresses questions about social life that require children or adolescents to coordinate different interests in dyadic relationships, the family, peer groups, and institutions. We consider social reasoning more complex than ToM and empathy (Nucci, 2019). Social reasoning often requires more than the capacity to understand and empathize with the other’s situation (*Nice is not enough*, Nucci, 2008). It includes the ability to engage in constructive conflict and argumentation and to question the status quo in power relationships and institutions that supports unfair practices and inequalities.

Lawrence Kohlberg’s cognitive developmental model postulates that the capacity to balance conflicting interests develops in an invariant sequence of six stages of moral judgment through childhood, adolescence, and adulthood (Kohlberg, 1975). The six stages are organized in three levels (each including two stages): the preconventional level (focus on sanction, obedience, and self-interest), the conventional level (focus on social norms and respect for law and order), and the postconventional level (focus on autonomous and principled moral judgments). According to Kohlberg’s stage theory, postconventional thinking does not develop before adolescence (if at all), as children and many adolescents or adults confound issues of justice and welfare with social expectations from groups or institutions (conventional level) or with personal interests or rights (preconventional level).

However, research from the perspective of Social Domain Theory (SDT) used a more suitable methodology to assess moral judgments (e.g., age-appropriate hypothetical vignettes), and more than 100 studies have revealed that preschoolers develop an emerging capacity for genuine moral judgments (Nucci, 2008; Smetana et al., 2014; Turiel, 1983, 2001). Three-year-old children judge hypothetical straightforward moral transgressions as wrong independently from authority, rules, and context. For example, they consider hitting wrong, even if it is allowed by teachers or parents and there are no rules against it. Moreover, they condemn hitting not only at school, but also in the family and in any context. In justification of their judgments, they refer to the intrinsic consequences of harmdoing for others’ welfare and rights and therefore reason about moral transgressions based on universalizable moral principles, such as fairness, welfare, and equality (e.g., “because it hurts,” and “it is unfair!”). In contrast, their judgments of social-conventional transgressions depend on authorities, rules, and context. For example, they consider it okay to address the teacher by his or her first name if the teacher agrees or if the school rules support it. Children also accept that forms of address might differ between schools and that school leaders have the authority to decide on these issues. In their justifications of judgments about social-conventional transgressions, they refer to the role of social norms for the smooth and effective functioning of social systems such as peer groups, families, or schools (e.g., “because there is a rule against it”; “it would get very chaotic”). Finally, children view personal issues (e.g., choice of friends or toys) as questions of free choice, personal preference, or privacy. They reject the notion that authorities have legitimate control over these issues (e.g., “it is my decision what to wear”). However, for prudential issues focusing on the possible negative effects of risky behaviors on a child’s welfare (e.g., issues of safety and health), younger children (but not adolescents) accept the control and restrictions of adults. Taken together, developmental researchers have shown that children develop an early capacity for conceptual distinctions between moral, social-conventional, and personal issues.

This certainly does not mean that young children are fully morally competent. Social domain theory explains development in two ways: First, children develop increasingly complex conceptions within each single domain (e.g., about distributive justice in the moral domain; Turiel, 1983). Second, with age, children and adolescents grow in their capacity for coordination of moral, social-conventional, and personal considerations that are sensitive to the specific context. While younger children reliably judge straightforward events in the three domains, older children and adolescents better recognize and balance various concerns in more ambiguous situations (Smetana et al., 2014). 

For example, complex situations of social exclusion require that children balance concerns about the harmful consequences of exclusion (moral domain) with concerns about effective group functioning or group identity (social-conventional domain) and individual sympathies for specific inclusion and exclusion targets (personal domain) (Killen & Rutland, 2011). With increasing age, children are more likely to consider the complexity of the exclusion context and prioritize moral, group, and personal concerns in a context-specific way (Gasser et al., 2014, 2017; Nucci & Turiel, 2009). Similarly, with increasing age, children are more sensitive to contextual issues in their judgments about deceptive behaviors. Older children are more likely to accept deception about noncompliance with directives from adults that are immoral (e.g., teacher directs a child to hit a peer) or personally intrusive (e.g., teachers direct a child to end a friendship; Gingo, 2017). This does not mean that older children doubt the generalizability of the honesty norm; it shows that compared with younger children, they understand that under certain conditions, other concerns should be prioritized over honesty. In sum, while ToM and empathy significantly contribute to basic social functioning, social reasoning requiring coordination of multiple concerns, such as criticizing personal interests or social conventions from a moral point of view, represents a more complex sociomoral competence.

## Three Conceptions of Sociomoral Learning Through Narrative Fiction

According to the transactional reading theory, reading reflects a dynamic and reciprocal relationship between the reader and the text (Chinn et al., 2001; Rosenblatt, 1978). Readers actively engage with texts by approaching them with different goals, referred to as *stances*, which affect how and what they learn from texts. Readers who approach a specific text from an efferent stance primarily focus on the extraction of information. In contrast, readers who approach the same text from an expressive stance focus their attention on the lived-through experience while reading. Finally, a critical-analytic reading focusses on more comprehensive and elaborative engagement with narrative fiction that essentially includes a critical evaluation of the initial responses to the text (Rosenblatt, 1995; Soter et al., 2010). These stances (efferent, expressive, and critical-analytic) have been effectively used to systematize various text-based discussion approaches (Murphy et al., 2009). We believe that the stances also provide a useful taxonomy to systematize different conceptions about sociomoral learning through narrative fiction, because Rosenblatt (1995) sees sociomoral development as an ultimate goal of the literary transaction and consistently evaluates the stances according to their contribution to this goal. However, the three stances are not exclusive. Readers can take a single specific stance, but more often various stances overlap. Similarly, the psychological and educational studies often include elements of several stances. Therefore, we have systematized the studies according to their dominant stance. As the selection of one or several stances often happens implicitly, a more intentional approach is more likely to lead to the desired developmental outcomes (Murphy & Firetto, 2017).

The research reviewed in the following sections assumes that specifically the *narrative fiction* genre, not just any text, can bolster readers’ sociomoral development. We define narrative fiction (e.g., short stories and novels) as texts that are structured around human goals and emphasize psychological experiences, social relationships, and social interactions (e.g., with protagonists acting on their main goals, experiencing conflicts with others’ interests, and resolving conflicts; Mar & Oatley, 2008). Therefore, narrative fiction, by definition, focuses on social content. In contrast, expository texts include more technical vocabulary and do not use human intentions as the organizing principle. We refer to *literary fiction* when researchers make specific claims about the impact of literary quality (e.g., outstanding language; Kidd & Castano, 2013). Table 1 summarizes the most important themes we abstracted from the literature and their integration within the theoretical framework of literary stances.

**Table 1:** Summary of the three conceptions of sociomoral learning through narrative fiction

Literary stances			
Themes	Efferent	Expressive	Critical-analytic
Learning mechanism	Direct instructions about “right” and “wrong” behavior through literary examples and models	Imagination of the perspectives and emotions of fictional characters	Argumentative dialogues with peers about initial responses to narrative fiction
Developmental outcomes	Accumulation of abstract moral propositions; culturally conforming social behavior	ToM, empathy	Social reasoning, interpersonal competences
Type of narrative fiction	“Classic” stories with straightforward moral messages	Narrative fiction of high literary quality (literary fiction)	Narrative fiction with complexity and ambiguity
Instructional principles	Strong didactic guidance toward a single "right" text interpretation	Eliciting spontaneous and individual responses to literary fiction through creative tasks and peer dialogues	Supporting argumentative dialogues with peers; critical examination of initial responses
Educational relationships	Asymmetric and hierarchical text-child and adult–child relationships; adults as moral experts, children as moral novices	Symmetric adult–child relationships; peers as important source for sociomoral learning	Symmetric and flexible power relationships in the classroom; peers as important source for sociomoral learning

### Efferent Approaches: Getting the Lesson

From the efferent stance, the reader’s “(…) attention is primarily focused on selecting out and analytically abstracting the information or ideas or directions for action that will remain when the reading is over” (Rosenblatt, 1995, p. 32). The reader prioritizes the objective information from the text over his or her spontaneous responses to and personal connections with the text (Rosenblatt, 1978). From this perspective, narrative fiction is used instrumentally for sociomoral learning, that is, to transmit a moral message or to provide directions for social action. Developmental outcomes of successful transmission are indicated by (a) the cumulative acquisition of moral propositions and (b) the direct transfer of these propositions into children’s social behavior.

#### Psychological Assumptions: Direct Instruction

Approaches that see the educational function of narrative fiction in the transmission of moral lessons explain sociomoral learning by a unidirectional top-down socialization process. They conceptualize the relationship between the text and the child as asymmetric and hierarchical; moral truth lies within the text and is waiting to be reproduced by the morally novice child (Lapsley & Narvaez, 2006). In particular, the so-called “classic” texts (e.g., fables and myths) instruct readers about universal moral truths (Bennett, 1995; Carr & Harrison, 2015; Kilpatrick, 1993). Owing to the seemingly clear moral lessons of classic texts, it is assumed that all children incorporate the lessons in the same way (for a critical discussion, see Narvaez, 2002). Behavioral learning plays an important role in explaining the internalization of the texts’ moral messages (Hart et al., 2020; Yao & Enright, 2020). Classic stories instruct the reader directly by providing fictional models for virtuous behavior or by highlighting the immediate positive or negative consequences of morally right or wrong behavior (Arthur et al., 2014; Leming, 2000). Reasoning and argumentation play a minor role, because the source for the justification of moral values is located in the text itself and not in the reader (Jerome & Kisby, 2020).

However, the empirical evidence for the direct socialization effect of narrative fiction on sociomoral development is weak and inconsistent. Most studies have focused on the effects of narrative fiction on honesty and generosity in young children. Lee et al. (2014) investigated how three classic stories compared with a control story affected honesty in 3- to 7-year-old children after they were tempted to cheat in a guessing game while the experimenter was absent. One of the classic stories (*George Washington and the Cherry*) highlights the positive consequences of honesty (the father praises the child for being honest), while the other two stories focus on the negative consequences of lying (*Pinocchio* and *The* *Boy who Cried Wolf*). Only the story focusing on the positive consequences of honesty influenced children’s confessions in the experimental situation. However, other studies have either completely (Butean et al., 2020) or partially failed (Talwar et al., 2018) to replicate the positive effect of the story *George Washington and the Cherry* on children’s confessions. Research on children’s generosity (assessed by distribution tasks) also revealed inconsistent findings, with some studies showing that prosocial stories had no effect (e.g., Kruse et al., 2021) and other studies showing that only some stories (particularly highly simplified and realistic stories) had a positive effect (Du & Hao, 2018; Larsen et al., 2018; Rottman et al., 2020).

Our first explanation for these contradictory findings refers to young children’s difficulties in understanding the theme of narrative fiction (Narvaez, 2002). The theme describes a generalized declarative proposition abstracted from the particularities of a story, and it often takes the form of a moral judgment or message (e.g., “honesty is good”; Kurtz & Schober, 2001). Younger children have difficulty transcending superficial story details and focusing on the more abstract ideas of a story (Pelletier & Beatty, 2015; van den Broek et al., 2003). For example, Mares and Acosta (2008) showed 5-year-old children a film about the social exclusion of a three-legged dog, intended to transmit a moral lesson about tolerance toward persons with disabilities. More than 80% of the children picked themes from a list that either simply summarized the story plot or described an irrelevant moral lesson. In a study by Narvaez et al. (1999), third graders, fifth graders, and adults read narrative fictional texts that included lessons about fairness and then evaluated their similarity with four short vignettes that were systematically varied according to deep and surface characteristics (e.g., setting, figures, plot, or theme). Only 11% of the third graders, 45% of the fifth graders, and 91% of the adults selected the vignette entailing the same theme. Third graders most often selected the vignette with the same plot (but a different theme), while fifth graders were less affected by superficial details. Therefore, the understanding of the theme of a narrative text represents a complex task, which younger children find more difficult to resolve than do older children. Narvaez (2002) concluded that narrative fiction does not speak directly to children such that all children understand the same lesson as intended by the author. Owing to the constructive nature of the reading process, the learning outcome of a reading event strongly varies as a function of reader characteristics (e.g., children’s cognitive or social skills, personal and cultural experiences) and reading context (e.g., instructional setting).

A second explanation for the inconsistent findings relates to the contextualized and reflective nature of sociomoral action (Turiel, 2001). Sociomoral actions are often complex, because they require balancing various moral, social-conventional, and personal concerns in a context-specific way. Therefore, sociomoral actions do not represent habitual reactions to any type of situation. The abstract moral messages of narrative fiction are notoriously subcomplex compared with the ambiguity of many real-life sociomoral conflicts. For example, in the study by Talwar et al. (2018), the children played with a first experimenter who pretends to accidentally break a toy and asks the child to keep the secret about the incident from a second experimenter. When the second experimenter returns (and the first experimenter leaves), he or she reads *George Washington and the Cherry* and then asks the child what happened during his or her absence. A story that simply tells children that they can expect praise from adults for being honest is relatively poor advice for a complex decision between telling the truth and keeping a promise. Deciding about when honesty is appropriate requires flexible thinking that has little to do with the direct application of moral messages in children’s books. As outlined in the previous section, social reasoning development in childhood and adolescence is less about understanding basic moral or social norms and personal issues, but about coordinating and applying concerns in multiple domains in a way that is sensitive to the complexity of many social situations.

Moreover, we are unaware of any study that has investigated whether reading narrative fiction also relates to children’s social behavior outside an experimental situation. Therefore, the studies do not allow the conclusion that narrative fiction affects children’s behavior in many different contexts. Children who act in accordance with the stories’ moral messages might simply be motivated by conformity concerns within the experimental context. Finally, it must be questioned that narrative fiction is a primary source for the development of moral knowledge. Moral knowledge develops through children’s reflections about specific experiences in interaction with peers and adults in early childhood (Dahl & Campos, 2013; Smetana et al., 2014). Once developed, this knowledge is quite robust against counterintuitive direct instructions (Kim et al., 2016). The idea that classic stories teach children to distinguish “right” from “wrong” (Kilpatrick, 1993) ignores the moral competences that young children already have and bring into their transactions with narrative fiction. In sum, studies on the effects of classic stories on children’s moral behavior do not provide strong support for a direct socialization effect. Evidence that children easily internalize and transfer the text’s moral messages into real-life social behaviors is limited.

#### Educational Approaches

The efferent perspective is most dominant in approaches on traditional character education (Bennett, 1995; Carr & Harrison, 2015; Kilpatrick, 1993; Leming, 2000). Typically, these approaches justify the need for classic stories by citing their orienting function in times of general moral decay in youth. They assume that classic stories include a single and universally valid text interpretation and deemphasize controversial discussions about different interpretations in order to prevent moral relativism (Lewin, 2020). Examples of current literacy programs in traditional character education include the “Knightly Virtues Programme” (Arthur et al., 2014; Carr & Harrison, 2015) and the “Narnian program” (Francis et al., 2018; Hart et al., 2020). These programs use classic stories such as *El Cid*, *Merchant of Venice*, or the Narnian novels with the aim of motivating children to imitate the virtues of fictional heroes. The goal of these programs is “(…) to help students increase their understanding of the virtues exhibited by the characters in the assigned novel; to help students increase their behavioural application of the virtues in their own lives” (Francis et al., 2018). Hence, they focus on developmental outcomes typical for our characterization of the efferent approach: Students learn from classic stories mainly by extracting moral content and applying this knowledge in real-life situations.

Before reading, teachers provide children with general definitions of the virtues (e.g., justice, honesty, service, or self-discipline), which they next identify in the text (e.g., by marking text passages). Thus, the children’s attention is consistently oriented toward the text’s moral message. To provide straightforward illustrations of the virtues, these programs either use excerpts or rewritten versions of the original stories. In the “Knightley Virtues Programme,” children primarily work with their literacy journal and discussions are discouraged, because “journals provide a more personal and private space for such reflection that is not so readily available in the contexts of such more public activities” (Carr & Harrison, 2015, Location No. 1928). The teacher’s role in facilitating active engagement with narrative texts remains largely unspecified and it is assumed that children will arrive at the right conclusions by themselves (Jerome & Kisby, 2020). These programs mostly focus on upper elementary and secondary students, but also address younger children (e.g., Freeman, 2014).

Evaluation studies on literary-based character education programs mostly focus on student outcomes such as virtue knowledge (assessed by multiple-choice tasks about virtue definitions) or virtuous behavior (assessed by self-ratings on behaviors such as self-discipline). Overall, these studies showed that the programs helped students improve their virtue knowledge, but not their virtue behavior (e.g., Francis et al., 2018; Leming, 2000; Pike et al., 2021). Therefore, there is little evidence of a far-reaching effect of these programs on students’ sociomoral development. Moreover, the findings raise the question whether students build this knowledge simply based on teachers’ direct instruction on virtue vocabulary rather than by their engagement with the stories. It is unclear how literary programs that do little to actively engage students with the stories can support students’ deeper processing of the texts’ moral messages.

#### Conclusions

From the efferent perspective, the readers’ attention is primarily focused on abstracting the moral message from texts and transferring this knowledge to real-life situations. The goal of efferent educational approaches is to guide students toward an established and uncontested interpretation of the narrative fictional texts, typically classic stories. However, even seemingly simple classic stories (e.g., Aesop’s fables) invite a wide range thematic interpretations (Lewin, 2020). Traditional character educational programs strongly limit instructional activities (e.g., open peer discussions) that allow for multiple interpretations in order to provide straightforward moral guidance (Levine, 2019). Narvaez (2002) criticized the passive reader theory underlying these approaches, which not only deemphasizes children’s contributions, but also the teacher’s complex role in facilitating construction of meaning from texts. Moreover, most psychological research on effects of classic stories on social behavior focus on younger children, while most character education programs include early adolescents. Early adolescence marks a developmental period where students develop strong needs for freedom of choice in the personal domain (Nucci & Turiel, 2009; Smetana, 2011) and an increasing sensitivity to peer group norms in the social-conventional domain (Killen & Rutland, 2011), which both can conflict with moral concerns. Literary education that over-moralizes ignores opportunities to engage early adolescents in more complex thinking that integrates multiple perspectives and strives for well-balanced solutions (Rosenblatt, 1995).

The idea that readers accumulate abstract moral propositions by reading narrative fiction has also been criticized as an “acquaintance approach” (Carroll, 2000). This approach is problematic because the general moral messages of narrative fiction mostly describe truisms that—as developmental research has shown—are developed early in childhood and do not provide new moral insights even for younger children (Killen & Rutland, 2011). An exclusive focus on the moral message misses the real educational potential of narrative fiction for sociomoral learning. Narrative fiction does not provide new knowledge about right or wrong (“knowledge that”), but enables the reader, through the use of concrete and artful language, to imagine *how* it would feel to be in a specific situation (“knowledge how”) (Carroll, 2000). Carroll concludes:

For what art teaches us generally is not new maxims and concepts, but rather how to apply them to concrete cases, engaging and exercising our emotions and imagination, our powers of perceptual discrimination, moral understanding, and reflection, in ways that sustain and potentially enlarge our capacity for moral judgment (pp. 368–369).

In the next section, we argue that the moral messages children retain after reading is over are secondary compared to the training of social-cognitive and emotional skills as children imaginatively engage in the simulation of social worlds during reading.

### The Expressive Approach: Simulating Social Worlds

Rosenblatt defines this stance as the readers’ attention being “(…) centered directly on what he is living through during his relationship with that particular text” (Rosenblatt, 1978, p. 25). The readers’ primary concern is with the moment-to-moment perceptual, cognitive, and affective responses during the reading event. While the efferent reader socially learns from narrative fiction by extracting specific moral content from the text, the expressive reader develops mindreading and empathy by applying social-cognitive and affective processes while imagining the social worlds described in narrative fiction*.* From this perspective, the literary quality of narrative fiction plays an important role, because it stimulates the reader’s vivid imagination of particular cases and thereby cultivates *perception* (Nussbaum, 1985), that is, the reader’s deeper understanding of complex life circumstances.

#### Psychological Assumptions: Simulation of Social Worlds

Cognitive psychologists explain sociomoral learning through narrative fiction by a deeply embodied mental simulation of social worlds (Mar, 2018b; Oatley, 2016). Even preschoolers spontaneously imagine the mental states of fictional characters by taking their visual, spatial–temporal, and psychological perspectives and thereby experience it as if they were perceiving the situations directly (Fecica & O’Neill, 2010; Ronfard & Harris, 2014). Narrative fiction creates a kind of twin-world, similar to the mental models that emerge in children’s pretend play (Lillard, 2001). Readers absorbed in narrative fiction quarantine the imagined world from the real world by reducing cognitive efforts in questioning the reality status of narrative fictional contents (Prentice et al., 1997). They affectively and cognitively participate in the imagined world in a manner similar to their engagement in real-life contexts (Bezdek et al., 2013; Gerrig, 1993). Even though the setting, plot, and characters are fictional and decoupled from the real world, the complexities of social interactions and relationships in narrative fiction meaningfully relate to the real world (Mar & Oatley, 2008). As in real life, the mental states of fictional characters are often opaque, which requires readers to put themselves in their shoes and infer their intentions, emotions, and beliefs (Zunshine, 2006). Therefore, narrative fiction has been described as a *moral laboratory* that enables readers to have vicarious social experiences and exercise and expand their mindreading and empathic capacities (Hakemulder, 2000).

Over the last fifteen years, research on the effects of reading narrative fiction on ToM and empathy has strongly increased (for reviews, see Barnes, 2018; Mar, 2018a, b; Mar & Oatley, 2008). Most studies focused on adults and used the Reading the Mind in the Eyes test and the Interpersonal Reactivity Index to assess ToM or empathy. Reading habits were typically assessed by the Author Recognition Test (ART). This test requires participants to select names of authors of narrative fiction from a list and distinguish them from foils. The same procedure is used to recognize authors of expository texts. Several studies consistently revealed relations between lifetime exposure to narrative fiction and ToM and empathy (for a meta-analysis, see Mumper & Gerrig, 2017). In contrast, lifetime exposure to expository texts did not correlate or negatively correlate with sociomoral outcomes. Experimental studies further studied the effect of short-time exposure to narrative fiction (e.g., Kidd & Castano, 2019; Panero et al., 2017). Even though these studies produced less consistent findings, a recent meta-analysis has shown that short-time exposure might improve social-cognitive performance as well (for a meta-analysis, see Dodell-Feder & Tamir, 2018).

Less is known about the effect of the literary quality of narrative fiction on ToM and empathy. Some researchers have argued that narrative fiction entailing multidimensional fictional characters, ambiguities, and complexities challenges the reader to imaginatively engage more than narrative texts with flat characters and easily predictable story plots do (Kidd & Castano, 2013). Complex stories interrupt habitual reading and require the reader to engage with the text actively and creatively by filling in gaps and testing out multiple interpretations. It has also been argued that outstanding stylistic features of literary fiction (e.g., metaphors) support contemplative processes and allow for new psychological insights in readers (Koopman, 2015). However, the findings from experimental investigations are inconsistent. While some study findings revealed that one-time exposure to literary fiction had more of a positive effect on adults’ ToM compared with one-time exposure to popular fiction or nonfiction (Kidd & Castano, 2013), these outcomes did not emerge in other studies (e.g., Samur et al., 2018). Arguably, long-time exposure to high-quality literature is necessary to discover if there are reliable effects on readers’ ToM. Moreover, it is also possible that any kind of text stimulates readers’ sociomoral development, as long as the text is narrative (Mar, 2018b) and read with high imaginative and emotional engagement (Barnes, 2018).

To date, few studies have included children. Mar et al. (2010) showed that the performance of 4- to 6-year-old children on a battery of ToM tasks scaled by Wellman and Liu (2004) was significantly associated with children’s exposure to narrative fiction, assessed by parents’ recognition of authors or titles of children’s books. This correlation held even when parents’ income and children’s gender or vocabulary were partialed out. However, the correlational nature of this finding allows no conclusion about the causal direction of the relation between reading and sociomoral competence; in other words, reading narrative fiction might positively contribute to children’s sociomoral development, but it is also possible that children with more advanced sociomoral skills show higher interest in narrative fiction.

Moreover, a third variable might explain the relationship. Developmental research highlights the important role parents’ mental state talk during shared book reading plays in their children’s ToM (Adrián et al., 2005). A meta-analysis revealed that shared book reading is one of the most effective developmental contexts for the stimulation of ToM in younger children (e.g., more effective than reminiscing; Tompkins et al., 2018). Frequent and elaborated mental state talk between parents and children in shared book readings consistently predicts higher performance in ToM tasks. Longitudinal studies suggest a unidirectional relation, namely, that parents’ mental state talk predicts children’s ToM, and not the reverse (e.g., Taumoepeau & Ruffman, 2008). For example, Adrián et al. (2007) showed that mothers’ mental state talk in shared book readings predicted their 3- to 7-year-old children’s ToM-performance one year later, while the reverse relation was not supported. However, the study further showed that mothers did more than explore the inner life of fictional characters. When mothers explained the fictional characters’ perspective, they often simultaneously coordinated different points of view (e.g., self, child, and fictional characters). This suggests that sociomoral learning not only results by simulating fictional characters’ perspectives but also by contrasting and integrating the mental perspectives of different discussion partners. In sum, parents’ discursive behaviors during shared book readings provide children with a framework with which to *enter a community of minds* (Nelson et al., 2003)—an issue which will be elaborated later in the chapter on the role of a critical-analytic stance in sociomoral development.

#### Educational Approaches

The goal of educational approaches from the expressive stance involves enhancing children’s imaginative and emotional engagement with narrative texts to inspire rich simulations of fictional characters’ inner worlds and spontaneous affective and personal responses in readers (Henschel et al., 2016; Lysaker et al., 2011; Schrijvers et al., 2019b). Instructional activities for achieving this goal mainly include writing activities, literary discussions, and other creative activities (role-play, staged reading, and drawing).

Intervention studies with younger children mainly use adult–child or peer discussions about picture books to explore the perspective of fictional characters and support personal connection between the book themes and children’s life. The goal of the literary program *Relationally* *Oriented Reading Instruction* (RORI) is to provide “(…) readers with opportunities for forming vicarious relationships with characters by imagining their thoughts, feelings, and intentions” (Lysaker & Tonge, 2013, p. 635). In the first step, the teacher models the social imagination of fictional characters’ perspectives by using predefined connection stems (e.g., I wonder; this is making me feel). In the second reading, the teacher asks students questions focusing on the exploration of the fictional characters’ inner worlds. Children’s imaginings of the books’ social worlds are further supported by writing letters to characters. Literary quality plays an important role in the selection of the books (e.g., language that invites social imagination). RORI significantly enhanced ToM skills (e.g., RMET and faux pas) in second- and third-grade students with social difficulties (Lysaker et al., 2011). A literary program that similarly stimulates social imagination in younger elementary school children through literary fiction is *Reading and Feeling* (Kumschick et al., 2014). In contrast to Lysaker et al., this study additionally included a control group design. This two-month program covered eight units on topics such as masked or mixed emotions, using a children’s book selected for its high literary quality. An important goal of the program was to provide children with an embodied experience of emotions by providing many opportunities for staged readings. The intervention included open discussions about the feelings addressed in the narrative fiction, creative tasks (role plays, theater, reflections about emotional body experiences, and drawing scenes), and activities requiring attention to the text’s literary devices (e.g., by inventing rhymes). During discussions, children were encouraged to reflect on their personal experiences with specific emotions. The instructor sustained perspective-taking by explicitly commenting on the inner voices of the fictional characters. The study findings revealed that compared with children from the control group, those from the intervention group improved more in their emotion vocabulary and understanding.

Research on expressively oriented literary programs for adolescents also highlights literary quality of selected texts, the importance of personal and spontaneous responses to texts, and rich explorations of character’s inner psychological worlds (e.g., Eva-Wood, 2004). One of the most promising program *Transformative Dialogic Literature Teaching* (TDLT) uses nine short stories of high literary quality addressing issues of justice (Schrijvers et al., 2019a). TDLT follows a two-step instructional approach. Adolescents first received opportunities to express their personal and authentic responses to literature through writing tasks (“internal dialogues”) and then discuss their personal reading experiences with peers (“external dialogues”). The teacher supported the internal dialogues by using a think-aloud strategy that explicitly models how readers can spontaneously and personally respond to narrative fiction. Students then applied the strategies by annotating their initial responses while reading and reflecting on how specific literary devices might contribute to these responses. Explicit instructions for external dialogues included reflections on a video-recorded literary discussion among peers. Compared with students that receive regular literature teaching, students from the TDLT group improved in their self-reported insights into self and others (e.g., “Story reading offers me insight into what other people are like”), imagination of fictional social worlds (e.g., imagery, experience-taking, and sympathy for characters), and attention to outstanding language. Even though the primary focus of TDLT is expressive, it also includes elements of the critical-analytical stance (e.g., explicit instruction on discussions). Taken together, this intervention research shows that instructional activities from the expressive perspective effectively enhance perspective-taking and empathy in both children and adolescents (for a systematic review, see Schrijvers et al., 2019b).

#### Conclusions

In expressive reading, the reader’s attention is focused on his or her direct experiences in transacting with a particular text (Rosenblatt, 1978). Cognitive psychologists conceptualize these experiences as a simulation of fictional social worlds (Mar, 2018b; Oatley, 2016). Unlike efferent reading, expressive reading does not focus on social learning by acquiring content, but by training social-cognitive processes. Readers become immersed in the narrative fictional world and create experiences as if they were part of this world. Educational approaches from the expressive stance recommend creative activities and peer dialogues that support rich explorations of fictional characters’ inner lives and connections to children’s own feelings and experiences. Typically, these approaches strongly focus on the literary quality of narrative fiction as they assume that attention to artful language significantly contributes to social imagination.

The expressive stance also has limitations. According to Rosenblatt (1995), the initial responses of readers are often immature or biased and should be expanded by more elaborate and comprehensive reflections. Balanced text interpretations require that students critically question their subjective responses by engaging in dialogues with peers and cooperatively contrasting and examining different perspectives on the text. Even though educational approaches from the expressive stance strongly recommend peer dialogues, they include little information about the specific instructional framework (Schrijvers et al., 2019b). They mainly provide students with opportunities to express their personal connections to the text, with little attention to the critical examination of the validity of different literary responses.

Moreover, ToM and empathy represent basic and value-neutral sociomoral competences, which can be instrumentalized for both social and antisocial goals (Nucci, 2019). The capacity to take others’ perspectives and understand others’ emotional expressions or masked emotions essentially contributes to achieving moral goals, but similarly supports the effective and strategic manipulation of others for egocentric goals. Bloom (2017) further argued that empathy is often biased, because it directs our attention to the suffering of a single person, thereby obscuring our sound judgment of the needs of others (e.g., favoring individuals from the ingroup over individuals from outgroups). Empathy for victims can also function as a catalyst for anger and retaliatory desires toward offenders. Narrative fiction is a powerful vehicle for persuasion and therefore can simultaneously evoke empathy and hatred in readers (Bloom, 2010). While the expressive approach sees sociomoral learning as the result of narrative absorption and identification with fictional characters, this experience bears the risk of uncritically following the text’s underlying arguments (Prentice et al., 1997). Critical thinking and social reasoning aim to balance diverse perspectives rationally, thereby providing basic sociomoral competences with a responsible direction. Nussbaum eloquently concludes that “perception without responsibility is dangerously free-floating, even as duty without perception is blunt and blind. The right ‘basis’ for action is found in the loving dialogue of the two” (Nussbaum, 1985, p. 524). In the next section, we contend that classroom dialogues about narrative fiction need to fulfill specific characteristics if educators aim to promote more than basic sociomoral competences.

### The Critical-Analytic Approach: Dialogic Inquiry

The transition from initial responses to more balanced interpretations is a process by which children learn to reflect rationally on their emotions and reflexive judgments about a particular text. According to Rosenblatt (1978), the initial responses are a necessary but insufficient condition for intellectual and sociomoral growth. Once the students have responded freely, they can develop “(…) the mental habits that will lead to literacy insight, critical judgment, and ethical and social understanding” (p. 71). Nussbaum (1996) characterizes this stance toward narrative fiction with Adam Smith’s metaphor of the *judicious* *spectator*: The reader imagines the perspective of fictional characters vividly and empathically, but without becoming biased and abandoning a critical evaluation of the fictional characters, the text’s moral message, or his or her own spontaneous responses (*finely aware and richly responsible*) (Nussbaum, 1985). The critical-analytic stance goes beyond the text’s moral message (efferent) and the simulation of characters’ perspectives (expressive) by emphasizing reasoned and justified judgments about complex sociomoral issues.

However, reasoning actively includes an intersubjective dimension (Mercer, 2013; Nucci, 2019). Narrative fictional cases often include high complexity and ambiguity and therefore elicit various responses in different readers. Balanced text interpretations require that readers engage in argumentative dialogues by critically examining the validity of arguments underlying the various perspectives (Reznitskaya & Gregory, 2013). From the critical-analytic perspective, children learn socially from narrative fiction by engaging in argumentative dialogues that require them to reason for their own perspectives and consider divergent perspectives. Through argumentative dialogues with peers or adults, children learn to justify their interpretation, respectfully listen to others’ ideas, critically examine others’ arguments, and develop openness toward alternative and better justified positions (Murphy et al., 2014). Thus, literacy dialogues share similar characteristics with real-life dialogues about sociomoral conflicts. They similarly require that children resolve disagreements not strategically by power and persuasion, but by establishing mutual understanding and co-constructing well-justified solutions. Thus, literary dialogues create a social microcosm that provides children with opportunities to train dialogic and interpersonal competences essential for real-life sociomoral problem solving.

#### Psychological Assumptions: Dialogic Learning

Cognitive developmental approaches (including SDT) locate the mechanism for sociomoral development through narrative fiction in co-constructive and co-regulated literary dialogues between children, peers, and adults (DeVries et al., 2000; Kohlberg, 1985; Narvaez, 2001; Nucci & Ilten-Gee, 2021). In contrast to expressive reading, the critical-analytic approach stresses the importance of dialogues that include collaborative examination of text interpretations that go beyond expressing personal responses to the text. Critical engagement with the text counteracts the readers’ spontaneous tendency to concur with the beliefs implied by the narratives (Prentice et al., 1997). In contrast to efferently oriented approaches, critical-analytic approaches view children’s disagreements with and resistance to the author’s and co-readers’ positions as catalysts for individual and social change, and not as signs of children’s maladaptation or general refusal of moral standards. Disagreements require that children coordinate and integrate different perspectives on sociomoral issues to develop more equilibrated positions (Berkowitz & Gibbs, 1985).

Some studies have focused on the role parent–child conversations about picture books play in young children’s emerging capacity for critical-analytic reading and social reasoning. The use of socioemotional vocabulary and decontextualized language by parents and children positively contributes to children’s ability to distinguish moral from social-conventional transgressions (Aram et al., 2017). Similarly, parents’ explanations of moral rules and emotions during shared book reading predict higher moral reasoning levels in preschoolers (i.e., less hedonistic/sanction-oriented and more principled reasoning) (Chou et al., 2021). Even though these studies identified some early precursors of a critical-analytic reading, more research is needed to more comprehensively investigate how parents support young children in contextualizing and criticizing morally relevant actions of fictional characters (e.g., see Wainryb & Recchia, 2017, for research on parent–child conversations about morally relevant real-life narratives).

Typically, research on more complex forms of social reasoning relied on short fictional stories that present a moral dilemma. For example, children would be told a story about a class that aimed to share the money they earned by selling their drawings fairly, while classmates asserted different claims (i.e., strict equality, merit, and equity). The children and adolescents would then discuss with peers or parents how they would share the money and the reason for it. This research revealed that dialogues characterized by frequent transformation of others’ arguments (e.g., critiques or clarifications) more effectively stimulated children’s and adolescents’ social reasoning development than did dialogues that primarily represented the other’s perspective (e.g., by paraphrasing) (Berkowitz & Gibbs, 1985; Killen & Damon, 1982). This quality of dialogue is more pronounced in children’s dialogues with peers than dialogues with adults (Kruger, 1992; Kruger & Tomasello, 1986). For example, 4- to 6-year-olds more often spontaneously justify their opinions and challenge the others’ positions in dialogues with peers than in dialogues with mothers about a short picture book (Mammen et al., 2019). Children more quickly surrendered their position in dialogues with mothers, because they may have viewed their solutions as “non-negotiable dictums” (p. 2324). In contrast, in dialogues with peers, they may have experienced different perspectives as similarly legitimate, and they were therefore more likely to resolve the disagreement by argument. However, if adults share control with children and allow leadership by the child, adult–child dialogues are similar to peer dialogues in predicting social reasoning development as peer dialogues (Kruger, 1992). Finally, disagreements should not happen at the expense of the affective quality of dialogues. Dialogues characterized by high emotional support and collaborative norms positively contribute to children’s and adolescents’ social reasoning (Kruger, 1992; L. J. Walker et al., 2000). Therefore, cognitive challenges need to be balanced with supportive interactions (Allen et al., 1994; Killen & Damon, 1982).

Unfortunately, this research strongly relied on short narrative texts. Nevertheless, the narrative fiction genre might contribute children’s social reasoning more generally. Compared with talk about expository texts, talk about narrative fiction is characterized by more complex language and richer mental state talk (Nyhout & O’Neill, 2013). Moreover, the narrative fiction context requires children to construct a coherent account about fictional characters’ course of actions. For example, children need to carefully balance their empathic understanding of fictional characters’ actions (contextualizing) with considerations about the consequences of these actions for other’s welfare (moral critics; Rosenblatt, 1995). Although parent–child talk about real-life narratives (i.e., reminiscing) similarly contributes to this goal (Recchia et al., 2014), the narrative fiction context is unique because it illuminates hard life decisions in a highly coherent manner (Mar & Oatley, 2008). Therefore, conversations about narrative fiction might represent a privileged context for training of more complex forms of social reasoning (Mar et al., 2011).

#### Educational Approaches

Literary programs following the critical-analytic stance focus on the role argument-based peer dialogues about narrative fiction play in children’s sociomoral reasoning development. While many socioemotional intervention programs use language implicitly to promote social skills such as perspective-taking or empathy, critical-analytic literary programs strongly focus on language skills because “(…) by explicitly teaching children how to use spoken language more effectively, they will develop their empathetic capabilities and social confidence, as well as their thinking and reasoning skills” (Mercer et al., 2019, p. 298). In a meta-analysis, Murphy et al. (2009) identified text-based discussion approaches that contribute to peer-led dialogues and critical-analytic thinking more effectively than other discussion approaches do (e.g., Collaborative Reasoning, Philosophy for Children). These discussion approaches go beyond supporting basic text understanding and spontaneous responses to texts by including specific and explicit instructions on how to justify claims, extend others’ arguments, consider diverse perspectives, and respectfully challenge others’ ideas.

Although each discussion approach is unique, they share important similarities. Before the discussions, children learn about discussion rules explaining the normative expectations regarding the cognitive dimension (e.g., justify claims and consider alternative perspectives) and the social and affective dimension of dialogues (e.g., mutual respect and equal participation). Narrative fiction that raises complex questions about sociomoral issues is often used as a stimulus for argumentative peer dialogues. The dialogues often occur in small and heterogeneous groups of students. Professional development activities strongly focus on changing the teacher’s role during the dialogues. Instead of teachers structuring and dominating the dialogues, children take leadership and control over the form and content of the dialogues by speaking freely without raising their hands and without the teacher evaluating the students’ responses. The teacher actively takes a back seat during discussions, but supports the dialogues by *talk moves* (e.g., modeling, eliciting, and challenging). As children gain more practice, the teacher continuously releases responsibility to them. Children’s metacognitive strategies are supported by setting and reflecting on specific goals before and after the dialogues.

Several studies have revealed that critical-analytic instructional approaches support dialogues in which students assume authority over content and procedures, spontaneously initiate ideas, provide elaborative explanations, consider alternative points of view, and respectfully challenge peers’ arguments (Davies & Sinclair, 2014; Murphy et al., 2014; Topping & Trickey, 2014). Therefore, these instructional approaches systematically address the developmental processes relevant for children’s sociomoral reasoning development. However, most of the research focuses on the formal aspects of children’s dialogues and critical thinking, with only a few studies specifically investigating effects of literary dialogues on children’s social reasoning.

Walker et al. (2013) studied the effects of Philosophy for Children (P4C) on 7- to 8-year-old children’s thinking in the moral and non-moral domains (aesthetic, physical). Developmental progress in children’s social reasoning was assessed using an epistemological understanding task that required the children to grasp that although several perspectives on value conflicts are legitimate, not all of them are equally justifiable. Children in the P4C intervention group discussed several children’s books that raised controversial questions in moral and non-moral domains. Children from the control group participated in an art project that did not include any dialogic instructions. Children from the P4C group improved more than children from the control group did in their epistemological understanding of moral conflicts, while no difference was found between them in their understanding of non-moral issues. Moreover, children from the P4C group improved more in their capacity to produce arguments for and against their own position regarding conflicting issues.

Collaborative Social Reasoning (CSR) (Lin et al., 2019, 2021) is a six-week literary program that includes six to ten discussions about narrative fictional texts. The texts address provocative questions about friendships, social exclusion, ethics of care, and responsibility that should invite controversial discussions around a “big question.” The pre- and post-tests included a social reasoning essay task that required children to read a short narrative fictional text and then write an essay in response to a question about an ambiguous and complex moral decision. The findings revealed that children in the CSR group used more arguments in the moral, social-conventional, and personal domains and more often took the cognitive perspective of fictional characters than did children in the control group (Lin et al., 2019).

Microanalytic analyses further highlighted the important role of teacher scaffolding in the quality of students’ literary dialogues. The teachers often initiated social reasoning in children by using specific talk moves (e.g., eliciting evidence), but reduced their scaffolding when students autonomously engaged in CSR (Nagpal, 2022). Thus, minimal teacher interventions sufficed to encourage children’s independent use of social reasoning strategies (also referred to as “snowball phenomenon”; Lin et al., 2015). However, teachers not only engaged in cognitive scaffolding, but also managed group dynamics during the literary dialogues. Kraatz et al. (2020) showed the strong interdependence between cognitive depth of interpersonal social reasoning and the affective quality of group dynamics (e.g., high regard for others’ perspectives and group members’ equal access to the conversational floor). Therefore, effective scaffolding of intellectual depth requires that teachers be attentive to the social group dynamics, wherein students compete by pushing their own arguments, show little respect for other options, and frequently interrupt. Nonetheless, if teachers successfully balance the cognitive and social dimensions of group dialogues, they not only support the development of students’ social reasoning, but also their interpersonal negotiation strategies, which transfer to children’s social life in the classroom. Accordingly, Lin et al. (2021) showed that compared with children in the control group (read-aloud and regular instruction), children participating in the CSR group were accepted more by peers and showed less peer-nominated aggressive behavior.

#### Conclusions

From the critical-analytic perspective, sociomoral learning is explained by children’s engagement in argumentative dialogues about narrative fiction with peers, parents, or teachers. These studies focus on more complex social competences, such as children’s ability to engage in critical social reasoning. In contrast to the efferent approach, critical-analytic approaches conceptualize the relationship between child and teacher as non-hierarchical, bidirectional, and dynamic. Children and teachers share authority over the form and content of discussion and flexibly negotiate and adapt power relationships in the classroom. Teachers encourage children to critically question the authors’ moral messages as well as their own and peers’ interpretations of the texts. Critical-analytic discussion approaches assume that narrative fiction inheres multiple meanings. They avoid the traditional format of recitation that guides students toward receiving a specific moral lesson. In contrast to the expressive stance, from the critical-analytic perspective, not all sociomoral perspectives on narrative fiction are equally valid and justifiable. In dialogues with peers, children elaborate, critically question, and extend personal and initial responses to narrative fiction within respectful and emotionally supportive social relationships. The teachers’ role in these peer dialogues is complex, because they must simultaneously scaffold inclusive or cooperative group norms and cognitively rich dialogues. They should avoid dominating the dialogues and should provide the students enough opportunities for interpretative authority and leadership over the text and the discussion. Therefore, they should support the discussions as few as possible and as much as necessary. Typically, these approaches focus on narrative fiction that raises controversial issues, which elicit disagreements in peer dialogues. They seldom refer to literary quality of these texts.

A major limitation of the critical-analytic perspective on sociomoral learning through narrative fiction is the insufficient evidence base. Very few studies have specifically investigated the effects of argumentative dialogues on children’s and adolescents’ social reasoning. For example, the research cited above focused exclusively on elementary grade students and we do not know if a critical engagement with narrative fiction also positively contributes to social reasoning development in younger children. Moreover, critical-analytic approaches are susceptible to the instrumental use of narrative fiction for promoting children’s sociomoral development. Critical-analytic discussion approaches do not strongly recommend instructional activities aimed at enhancing children’s attention to outstanding language or intimate relationships with fictional characters, as do expressively oriented literary programs such as “Reading and Feeling” (Kumschick et al., 2014) and TDLT (Schrijvers et al., 2019a). As expressively oriented literary tasks (e.g., creative tasks) positively contribute to students’ interest in and emotional engagement with literary fiction (Henschel et al., 2016), it is unclear if instructional approaches including fewer of these tasks similarly enhance students’ motivation for literary education.

## Summary and Future Directions

The major goal of this integrative review was to systematize the literature on sociomoral learning through narrative fiction across different research communities that largely work separately from each other. We abstracted themes from the literature that characterize the implicit assumptions and educational principles of various approaches and organized them within a broader theoretical framework of literary stances (for the summary, see Table 1). The integrative review contrasts three fundamentally different conceptions about sociomoral learning through narrative fiction (efferent, expressive, and critical-analytical). We also included minority positions. Most psychological and educational research focuses on the expressive perspective (and to a lesser degree, the efferent perspective); very few studies have been conducted on sociomoral learning through narrative fiction from a critical-analytic perspective.

Our synthesis revealed that the expressive and critical-analytic approaches to literary education are the most promising for supporting children’s development of sociomoral competences. However, the analyses of the research literature showed that the two perspectives have very different goals: While expressively oriented approaches focus more on basic social skills, such as perspective-taking and empathy, critical-analytic approaches address more complex social competences, such as social reasoning and argumentative skills. The capacity to question social norms and practices essentially contributes to reflective democratic participation (Nussbaum, 2002). Therefore, research on literary education should extend the focus from social imagination to critical inquiry of ethical questions raised in narrative fiction texts.

Although the focus of this integrative review was the comparison of the three stances, we do not consider them independent from each other. The development of more complex forms of social understanding requires that first children vividly imagine and empathize with the perspectives of fictional characters (Nussbaum, 2002; Rosenblatt, 1995). Similarly, social reasoning about narrative fiction is not without content. If children critically examine the moral lessons of narrative fiction by juxtaposing different interpretations of the stories’ themes, such dialogues might significantly contribute to children’s social reasoning. Therefore, children’s thinking about the text’s moral messages and spontaneous responses built the raw material for a more reflective and critical engagement with narrative fiction. While expressive approaches include few opportunities for training argumentative dialogues, critical-analytic approaches pay little attention to the simulation of social worlds and the role of outstanding language. An important avenue for future research is to develop and test literary programs that give prominence to critical-analytical goals supported by the exploration of the text’s moral messages and emotive connections to the text.

This integrative review also attempted to bridge psychological and educational literature. Such integration not only helps clarify the implicit psychological assumptions of literary programs, but also highlights the importance of developing psychologically informed literary education programs. To stimulate children’s sociomoral development effectively, literary programs need to address the psychological processes assumed to underlie the development of specific sociomoral competences. For example, the developmental literature on the role of talk during shared book readings for children’s social understanding (e.g., Tompkins, 2018) might importantly inform the instructional design of literary dialogues in preschool classrooms. Moreover, the psychological research also contributes to the conceptual and empirical foundation of educational programs by providing precise definitions of developmental outcomes (e.g., morality) and reliable and valid assessments for efficacy studies (e.g., coding systems for analyzing morally relevant talk; Recchia et al., 2014). Therefore, we believe that future intervention research might benefit from a stronger interdisciplinary approach.

We used the transactional theory of literary stances for systematizing the literature on sociomoral learning through narrative fiction. Our characterization of the stances parallels distinctions of other important psychological models. For example, Mar (2018a) developed the *Social Processes and Content Entrained by Narrative* (SPaCEN) framework that similarly differentiates between extracting social information from narrative fiction (efferent) training social-cognitive processes (expressive). Krettenauer (2004) introduced three modes of metaethical cognition that highly parallel with the three conceptions of sociomoral learning through narrative fiction we outline in this article. From the intuitionist stance, morality is conceptualized as an objective reality and experts have privileged access to this reality. Similarly, from the efferent stance classic stories present universal moral truths that need to be internalized by the morally novice child. From the subjectivist stance, morality is viewed as a highly personal matter. This parallels the focus of the expressive stance on personal reactions to literary texts with little attention to the validity of these responses. Finally, the transubjectivistic stance balances these two stances by suggesting that although moral problems can be framed from multiple viewpoints, not all perspectives are equally justifiable. As suggested by the critical-analytic stance, the validity of various perspectives on narrative fiction needs to be established in an interpersonal evaluative process.

Historically, social interventions were designed as extracurricular programs that exclusively focus on social learning goals. Recently, researchers have increasingly highlighted the importance of developing interventions that naturally use opportunities for social learning in subject matters. It has been argued that integrative programs are more responsive to the interdependence of social and cognitive learning processes (e.g., Gasser & Althof, 2017; Jones et al., 2011). Literary education represents a promising context for systematically connecting social and academic learning. Literary education not only contributes to children’s language development, but also enhances children’s ability to imagine how it would feel to be a different person and develop interest in more distant lives. Future research should explore more comprehensively the extent to which literary education also supports children’s ability to question the status quo and critically reflect on complex social issues. In times of global social injustice and conflicts, these competences are more important than ever before.

## Data Availability

Not applicable.
